# Liver Enlargement Predicts Obstructive Sleep Apnea–Hypopnea Syndrome in Morbidly Obese Women

**DOI:** 10.3389/fendo.2018.00293

**Published:** 2018-06-06

**Authors:** Giovanna Scartabelli, Giorgia Querci, Letizia Marconi, Giovanni Ceccarini, Paolo Piaggi, Paola Fierabracci, Guido Salvetti, Giovanni Cizza, Salvatore Mazzeo, Jacopo Vitti, Slava Berger, Antonio Palla, Ferruccio Santini

**Affiliations:** ^1^Obesity Center, Endocrinology Unit, University Hospital of Pisa, Pisa, Italy; ^2^Pulmonary Unit, University of Hospital of Pisa, Pisa, Italy; ^3^National Institute of Diabetes and Digestive and Kidney Diseases, National Institutes of Health, Phoenix, AZ, United States; ^4^Eunice Kennedy Shriver National Institute of Child Health and Human Development (NICHD), National Institutes of Health, Bethesda, MD, United States; ^5^Department of Radiology, University of Hospital of Pisa, Pisa, Italy; ^6^Division of Pulmonary and Critical Care Medicine, Department of Medicine, The Johns Hopkins University School of Medicine, Baltimore, MD, United States

**Keywords:** non-alcoholic fatty liver disease, obstructive sleep apnea–hypopnea syndrome, hepatic left volume, metabolic syndrome, insulin resistance, morbid obesity

## Abstract

Obstructive sleep apnea–hypopnea syndrome (OSAHS) is frequently present in patients with severe obesity, but its prevalence especially in women is not well defined. OSAHS and non-alcoholic fatty liver disease are common conditions, frequently associated in patients with central obesity and metabolic syndrome and are both the result of the accumulation of ectopic fat mass. Identifying predictors of risk of OSAHS may be useful to select the subjects requiring instrumental sleep evaluation. In this cross-sectional study, we have investigated the potential role of hepatic left lobe volume (HLLV) in predicting the presence of OSAHS. OSAHS was quantified by the apnea/hypopnea index (AHI) and oxygen desaturation index in a cardiorespiratory inpatient sleep study of 97 obese women [age: 47 ± 11 years body mass index (BMI): 50 ± 8 kg/m^2^]. OSAHS was diagnosed when AHI was ≥5. HLLV, subcutaneous and intra-abdominal fat were measured by ultrasound. After adjustment for age and BMI, both HLLV and neck circumference (NC) were independent predictors of AHI. OSAHS was found in 72% of patients; HLLV ≥ 370 cm^3^ was a predictor of OSAHS with a sensitivity of 66%, a specificity of 70%, a positive and negative predictive values of 85 and 44%, respectively (AUC = 0.67, *p* < 0.005). A multivariate logistic model was used including age, BMI, NC, and HLLV (the only independent predictors of AHI in a multiple linear regression analyses), and a cut off value for the predicted probability of OSAHS equal to 0.7 provided the best diagnostic results (AUC = 0.79, *p* < 0.005) in terms of sensitivity (76%), specificity (89%), negative and positive predictive values (59 and 95%, respectively). All patients with severe OSAHS were identified by this prediction model. In conclusion, HLLV, an established index of visceral adiposity, represents an anthropometric parameter closely associated with OSAHS in severely obese women.

## Introduction

Obstructive sleep apnea–hypopnea syndrome (OSAHS), an emerging public health issue, is characterized by recurrent episodes of upper airway occlusion during sleep, which results in reduction or cessation of the airflow, and lead to chronic intermittent hypoxia and sleep fragmentation (1). The pathogenetic factors of OSAHS are manifold and yet not completely understood. The main cause seems to be an anatomical upper airway narrowing, where the increased negative intrathoracic pressure during inspiration exceeds the counteracting forces of the dilating muscles (2–4). The cyclical recurrence of obstructions and arousals may cause instability of central respiratory motor output, thus contributing to the genesis of apneic episodes (5).

Obstructive sleep apnea–hypopnea syndrome affects a significant proportion of the adult population, mainly males, and its prevalence increases with increasing body mass index (BMI) and advancing age (1, 6, 7). Currently, in the United States, among adults, approximately 13% of men and 6% of women have moderate-to-severe OSAHS [apnea/hypopnea index (AHI) ≥ 15] while 14% of men and 5% of women have an AHI ≥ 5 together with symptoms of daytime sleepiness (1, 8). The mechanisms linking obesity to OSAHS include pharyngeal narrowing due to fatty tissue in the lateral airway walls, muscle functional impairment due to fatty deposits, enlargement of the abdomen resulting in reduced lung volumes, decreased longitudinal tracheal traction forces and increased tendency of pharyngeal collapse during inspiration, the low-grade systemic inflammation associated with obesity (3, 9).

On the basis of animal models (10), it has been postulated that leptin resistance developed by obese patients may impair the neuroanatomic interaction necessary for stable breathing, thereby contributing to the genesis of OSAHS (11). The disruption of normal sleep and chronic intermittent hypoxia starts a vicious circle by worsening obesity and may explain the close association between OSAHS and some of the features of the metabolic syndrome (MS), including hypertension, insulin resistance, and type 2 diabetes, ultimately leading to cardiovascular and cerebro-vascular illness (12–15). The name “syndrome Z” has been proposed for the association between MS and OSAHS (16) and the inclusion of this sleep and breathing disorder among the manifestations of MS has been also suggested (17).

The Cardiometabolic Think Tank convened on June 20, 2014 in Washington, DC, has tried to categorize subtypes and stages of MS in order to find an optimal care model for patients at increased cardiometabolic risk. The subtype with the excess visceral adipose tissue as main pathophysiologic mechanism is characterized by the presence of sleep disordered breathing and non-alcoholic fatty liver disease (NAFLD) (18).

Patients with MS frequently have an increased fat (triglyceride) accumulation in the liver, called NAFLD, and hepatic insulin resistance. With the increasing worldwide prevalence of obesity, NAFLD has become the most common cause of abnormal liver function in the general adult population (19–23). Studies conducted in humans and mice have suggested that OSAHS could be a detrimental factor potentially responsible for the exacerbation of liver injury in obesity (3).

The diagnosis of NAFLD is usually established by ultrasound and can be confirmed by liver biopsy (24).

We have shown that the ultrasound measurement of the hepatic left lobe volume (HLLV) correlated with the total volume of liver measured with MR and is an excellent indicator of visceral adiposity clustering with parameters defining MS (25, 26).

Little is known about the prevalence of OSAHS and the impact of this medical disorder on metabolic risk factors in morbidly obese patients; especially in women in whom it frequently remains underdiagnosed (27).

The aim of this cross-sectional study was to evaluate in a group of morbidly obese women with symptoms and signs associated with OSAHS the prevalence of OSAHS and the relationships between the severity of OSAHS and several anthropometric measurements with particular interest to HLLV.

## Materials and Methods

This is a retrospective study that included 97 morbidly obese women (BMI > 35 kg/m^2^) referred to our Obesity Center for evaluation of obesity and its comorbidities and had, therefore, been submitted to cardio-respiratory sleep study for the clinical suspicion of OSAHS (excessive daytime sleepiness, choking or gasping during sleep, recurrent awakenings from sleep, impaired concentration). All patients signed a written consent for the treatment of their clinical data for purpose of research. The study did not require additional testing beside the protocol for evaluation of patients candidate to bariatric surgery. Exclusion criteria for this study were: history of hypothalamic–pituitary diseases, hypercortisolism, goiter, self-reported daily alcohol consumption > 20 g, current or past use of illicit drugs or hepatotoxic medications, viral hepatitis as assessed by conventional serum markers, a previous diagnosis of a disease potentially causing liver enlargement (e.g., storage diseases, autoimmune hepatitis), pregnancy or breast feeding within the 12-month period before enrollment. A careful endocrinological evaluation was also performed to reveal undiagnosed dysfunctions that required specific therapy and to evaluate the presence of hormonal abnormalities associated with OSAHS. 33 subjects were taking hypoglycemic agents, 7 hypolipidemic agents, and 4 hypouricemic agents.

### Anthropometric, Clinical, and Laboratory Measures

Clinical, hematological, and instrumental examinations were performed according to the Italian guidelines for obesity (28). Anthropometric measures were determined in the morning after an overnight fast. Body weight was measured using a stand-on-scale in a hospital gown to the nearest 1/10th of a kilogram (SECA gmbh & co. —Germany) and height was measured to the nearest centimeter using a wall-mounted stadiometer (Health o meter.inc., Bridgeview, IL, USA). Neck circumference (NC) was determined at the level of the cricothyroid membrane. Blood pressure on admission was recorded with a large cuff while the patient was recumbent. Venous blood was obtained after an overnight fasting for measurement of glucose, triglycerides (TG), high-density lipoprotein (HDL), low-density lipoprotein (LDL) and total cholesterol, uric acid, aspartate aminotransferase (AST), alanine aminotransferase (ALT), γ-glutamyltransferase (γ-GT), and alkaline phosphatase (ALP). The homeostasis model of insulin resistance (HOMA) was calculated based on fasting serum glucose and insulin concentrations (29). In 64 patients who were not taking hypoglycemic agents, an oral glucose tolerance test was performed in the morning with measurement of serum insulin and glucose when fasting, and every 30 min for 3 h after the ingestion of the glucose load (75 g).

### HLLV and Abdominal Fat Measurements

Ultrasound examination for measurement of HLLV, subcutaneous fat (SCF) and intra-abdominal fat (IAF) was performed, as previously described (25, 26). Briefly, the ellipsoid formula (width × height × length × 0.52) was employed to calculate the HLLV. Thickness of the SCF was taken 1 cm over the transversal umbilical vein, by measuring the distance between the skin and the external face of the muscular fascia, while IAF thickness was defined as the distance between the internal face of the same muscle and the anterior wall of the aorta. Abdominal thickness (AT) was defined as the distance between the skin and the anterior wall of the aorta.

### Sleep Apnea Assessment

An inpatient cardiorespiratory overnight sleep study was performed by means of a polygraph (Somno Check; Vivisol). To consider a study valid, a minimum sleep duration of 5 h was required. Parameters measured included oronasal flow by nasal cannula, thoraco-abdominal movements, pulsoxymetry, snoring, and body position. The results recorded by the instrument were scored by the attending physician expert in sleep studies who was maintained blinded to the characteristics of the patient. Nocturnal O_2_ saturation (SpO_2_) was recorded during the entire length of the night. Apnea was defined by the absence of airflow for > 10 s while hypopnea was defined as any airflow reduction of >50% that lasted for >10 s and resulted in oxyhemoglobin desaturation (3% dip rate). AHI was defined as the sum of the numbers of apnea and hypopneas per hour of sleep and OSAHS was diagnosed when AHI was ≥ 5. Oxygen desaturation index (ODI) was defined as the number of desaturations per hour of sleep and OSAHS was diagnosed when ODI was ≥5.

According to the American Sleep Disorders Association Task Force criteria (30), sleep-related obstructive breathing events were scored as mild when between 5 and 15 events/h of sleep, as moderate when between 15 and 30, and as severe when they were >30 events/h of sleep. Patients with a negative cardiorespiratory sleep study showing diurnal sleepiness underwent a complete polysomnography to avoid false-negative diagnosis in accordance with the American Sleep Disorders Association report (31).

### Statistical Analysis

The sample size calculation was based on the number of patients needed to demonstrate an association between HLLV and AHI. A target sample size of 84 patients provided a power of 0.80 with an alpha of 0.05 to detect a modest correlation equal to 0.30. With 97 patients recruited in this study, the statistical power was equal to 0.85. Descriptive statistics were calculated for the cohort as a whole and then separately by OSAHS diagnosis. Statistical tests used to compare groups included Student’s *t* test and ANOVA for difference in mean values, Mann–Whitney *U* and Kruskal–Wallis tests for skewed variables, Pearson Chi-square test for difference in counts and frequency. The Levene’s test was used to assess the equality of variances between OSAHS groups. The Kolmogorov–Smirnov test was used to assess normality of data; logarithmic transformations were applied to skewed variables (AHI, insulin concentrations and HOMA index) to approximate a Gaussian distribution. Pearson (R) and Spearman (Rho) correlation coefficients were employed to quantify associations for Gaussian and skewed variables, respectively. Multiple linear regression analyses using the forward selection algorithm were carried out to identify the most significant anthropometric predictors of AHI, SpO_2_, and ODI.

The receiver operating characteristic (ROC) curve was calculated to identify the cutoff value of both HLLV and NC, which better discriminated between subjects with and without OSAHS in terms of highest combined sensitivity and specificity (i.e., highest Youden index). The positive group included subjects with OSAHS (AHI ≥ 5) while the negative group those without OSAHS (AHI < 5). Sensitivity was defined as the percentage of subjects having OSAHS who were correctly classified as having this disease, while specificity was the percentage of subjects without OSAHS who were correctly classified as not having this disease.

A multivariate logistic analysis including all the determinants of OSAHS as determined through univariate analysis was carried out to calculate the predicted probability of OSAHS diagnosis (AHI ≥ 5) based on the logistic formula: 1/[1 + exp(−SCORE)], where SCORE was the linear combination of logistic model coefficients multiplied for the values of the respective determinant of OSAHS. A ROC curve analysis was then performed to determine the best cutoff value of the predicted probability of OSAHS diagnosis according to the highest Youden index, as above.

A *p*-value < 0.05 was considered statistically significant. Data are presented as a mean ± SD or median with inter-quartile range (IQR), as indicated. Statistical analyses were performed in SPPS (version 25; Armonk, NY, USA: IBM Corp.Armonk, NY, USA: IBM Corp.).

## Results

Average age of the study cohort (±SD) was 47 ± 11 years (range: 24–67) and BMI was 50 ± 8 kg/m^2^ (range: 36–81). The physical, hematological, and clinical characteristics are reported in Table 1. OSAHS was found in 70 patients (median AHI = 14, IQR: 8–25). Of all subjects with OSAHS, 53% had a mild OSAHS, 26% moderate OSAHS, and 21% severe OSAHS. Average SpO_2_ was lower in patients with OSAHS compared to those without (*p* < 0.005). Median ODI was higher in patients with OSAHS compared to those without (*p* < 0.001). On average, patients with OSAHS were 8-year older (*p* < 0.005) and had greater IAF and HLLV compared to patients without OSAHS (both *p* < 0.05) despite similar body weight, BMI, and SCF. In addition, higher levels of γ-GT were observed in patients with OSAHS (*p* < 0.05), and there was a positive association between AHI and γ-GT (Rho = 0.21; *p* < 0.05). No significant associations were observed between AHI and ALT (*p* = 0.21), AST (*p* = 0.22) or ALP (*p* = 0.49). Out of 70 patients with OSAHS, 48 (69%) and 50 (71%, *p* < 0.05) patients were classified as with the MS according to ATP-III and IDF criteria, respectively.

**Table 1:** Demographic and anthropometric (A), clinical (B) and laboratory characteristics (C) of the study population.

	All subjects (*N* = 97)	Without obstructive sleep apnea–hypopnea syndrome (OSAHS) (*N* = 27)	With OSAHS (*N* = 70)
**A**			
Age (years)	46.6 ± 10.7	40.8 ± 9.5	48.8 ± 10.3*
Body weight (kg)	126.9 ± 20.9	125.1 ± 16.8	127.6 ± 22.3
Body mass index (kg/m^2^)	49.6 ± 7.5	48.5 ± 6.0	50.0 ± 8.1
NC (cm)	38.4 ± 3.2	37.5 ± 2.7	38.7 ± 3.3
Subcutaneous fat (mm)	41.6 ± 14.2	41.6 ± 12.9	41.6 ± 14.8
Intra-abdominal fat (mm)	101.1 ± 32.6	90.3 ± 20.0	105.7 ± 35.8*
Abdominal thickness (mm)	142.1 ± 34.6	130.0 ± 21.1	147.3 ± 37.9*
Hepatic left lobe volume (cm^3^)	448.0 ± 242.7	344.6 ± 144.5	487.9 ± 261.3*

**B**			
Metabolic syndrome (%)			
ATP III criteria	62.9%	48.1%	68.6%
IDF criteria	64.9%	48.1%	71.4%*
Apnea/hypopnea index (AHI) (events/h)	18.5 ± 25.4	2.5 ± 1.6	24.6 ± 27.6*
Median [interquartile range (IQR)]	8.3 (4.7–18.4)	2.2 (1–4)	14 (8–25)*
AHI < 5 (no OSAHS)	27 (28%)	27 (100%)	0 (0%)
AHI ≥ 5 and <15 (mild)	37 (38%)	0 (0%)	37 (53%)
AHI 15–30 (moderate)	18 (19%)	0 (0%)	18 (26%)
AHI > 30 (severe)	15 (15%)	0 (0%)	15 (21%)
Mean nocturnal O_2_ saturation	92.7 ± 4.5	94.6 ± 2.9	92.0 ± 4.7*
Total sleep time (h)	8 ± 0.7	8.2 ± 0.7	8 ± 0.6
Oxygen desaturation index (events/h)	20.9 ± 24.1	7.0 ± 8.9	26.5 ± 26*
Median (IQR)	13.0 (5.4–23.0)	3.8 (1.3–7.8)	16 (10.2–29.6)*

**C**			
Fasting glucose (mg/dL)^a^	108.3 ± 45.3	102.2 ± 30.8	111.2 ± 51.0
2-h glucose (mg/dL)^a^	150.4 ± 74.9	145.2 ± 78.1	153.0 ± 74.2
Fasting insulin (μU/mL)^a^	13.3 ± 14.0	11.9 ± 11.2	13.9 ± 15.2
*Median (IQR)*	9 (6–14.5)	7 (5–13)	10 (6–16)
2-h insulin (μU/mL)^a^	73.0 ± 57.5	64.5 ± 48.2	77.4 ± 62.0
*Median (IQR)*	63 (34–88)	62 (32–88)	69 (41–89)
Insulin peak (μU/mL)^a^	98.3 ± 67.2	87.5 ± 71.2	103.9 ± 65.2
*Median (IQR)*	83 (59–113)	72 (45–107)	91 (64–116)
HOMA index^a^	3.7 ± 4.7	3.5 ± 4.9	3.9 ± 4.6
*Median (IQR)*	2.2 (1.4–3.7)	1.6 (1.3–2.8)	2.3 (1.6–3.9)*
Total cholesterol (mg/dL)^b^	195.5 ± 34.3	192.8 ± 40.1	196.6 ± 31.9
High-density lipoprotein (mg/dL)^b^	50.7 ± 15.9	51.4 ± 14.3	50.4 ± 16.7
Low-density lipoprotein (mg/dL)^b^	123.3 ± 29.9	116.3 ± 31.1	126.3 ± 29.2
Triglycerides (mg/dL)^b^	150.9 ± 67.8	146.1 ± 61.6	152.8 ± 70.5
Uric acid (mg/dL)^c^	5.6 ± 1.2	5.4 ± 1.1	5.7 ± 1.3
Aspartate aminotransferase (U/L)	23.8 ± 12.9	22.1 ± 13.4	24.4 ± 12.7
Alanine aminotransferase (U/L)	29.8 ± 16.8	27.0 ± 14.1	30.9 ± 17.8*
γ-glutamyltransferase (U/L)	35.8 ± 39.6	27.4 ± 23.4	39.0 ± 44.1*
Alkaline phosphatase (U/L)	125.7 ± 73.4	116.0 ± 74.8	129.4 ± 73.1

*Data are reported as mean ± SD, or frequency (%). For skewed variables, median values with IQR are also reported*.

*p < 0.05 vs. without OSAHS.

*^a^64 patients not taking hypoglycemic agents*.

*^b^90 patients not taking hypolipidemic agents*.

*^c^93 patients not taking hypouricemic agents*.

### Anthropometric Determinants of AHI and SpO_2_

**Figure 1:**
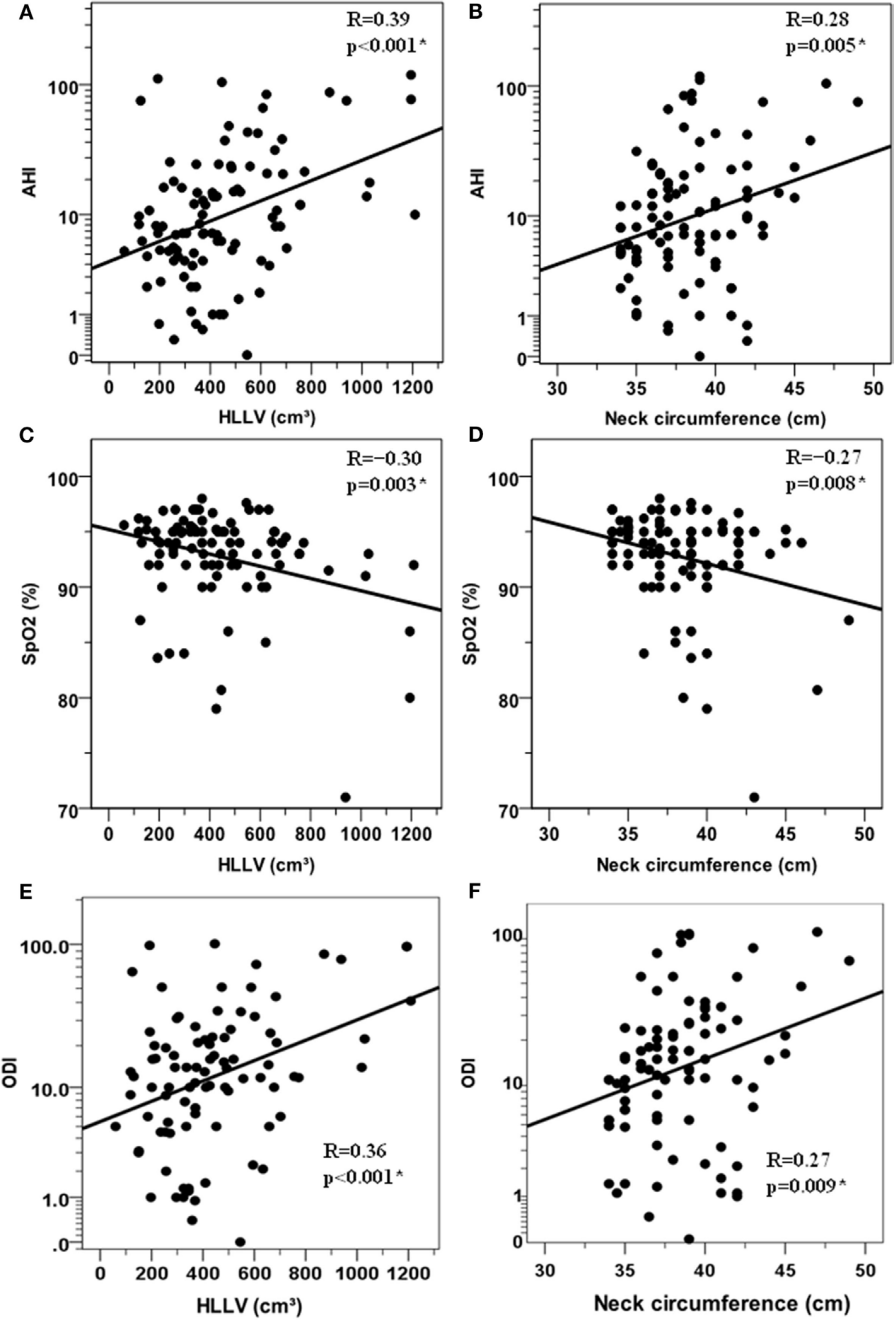
Correlations between anthropometric variables and respiratory paramenters in the cohort of 97 obese women. Relationships between the hepatic left lobe volume (HLLV) and the apnea/hypopnea index (AHI) **(A)** the mean percent oxygen saturation [SpO_2_, **(C)**] and the oxygen desaturation index (ODI) **(E)**. Relationship between neck circumference and AHI **(B)** mean percent oxygen saturation [SpO_2_, **(D)**] and (ODI), **(F)**. AHI and ODI values are reported on a logarithmic scale, i.e., LOG_10_(1 + AHI) and LOG_10_(1 + ODI) which can handle zero values. R: Pearson’s correlation coefficient (*p < 0.05).

**Figure 2:**
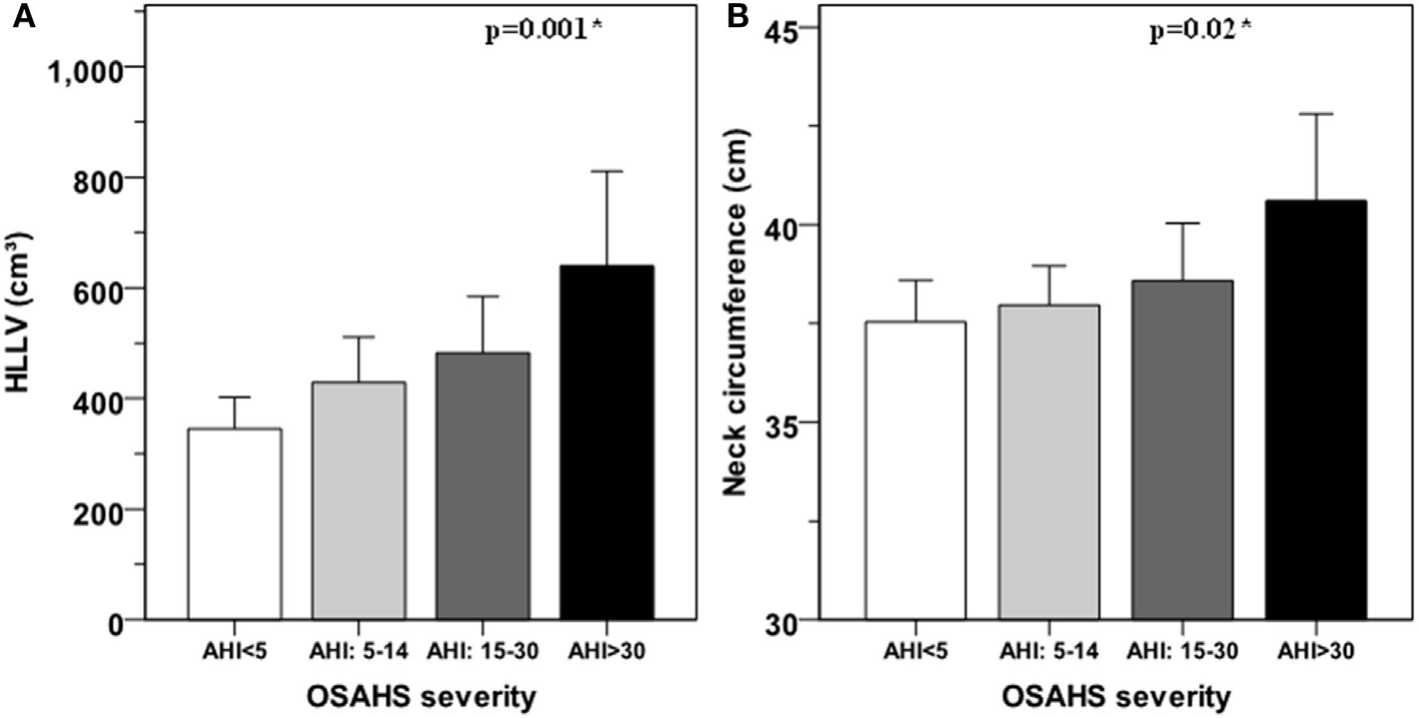
Hepatic left lobe volume (HLLV) **(A)** and neck circumference **(B)** average values for each class of obstructive sleep apnea–hypopnea syndrome (OSAHS). OSAHS severity is defined as mild for apnea/hypopnea index (AHI) ≥ 5 and < 15; moderate for AHI ≥ 15 and ≤ 30; and severe for AHI > 30. Error bars represent the 95% confidence interval of the mean. The variances of HLLV (*p* = 0.08) and neck circumference (*p* = 0.38) were not significantly different among OSAHS categories by the Levene’s test. *: *p* < 0.05 for linear trend by ANOVA.

Apnea/hypopnea index (Rho = 0.36, *p* < 0.005) and SpO_2_ (*R* = −0.30, *p* = 0.003) correlated with HLLV in a positive and negative fashion, respectively (Figures 1A,C). Similar associations with AHI (Rho = 0.24, *p* < 0.05) and SpO_2_ (Rho = −0.21, *p* < 0.05) were observed for NC (Figures 1B,D). ODI correlated with HLLV (Rho = 0.36, *p* < 0.001) and with NC (Rho = 0.27, *p* = 0.009) in a positive fashion (Figures 1E,F). Accordingly, both HLLV and NC increased with severity of OSAHS (trend *p* < 0.05, Figure 2). Further, IAF (AHI: Rho = 0.33, *p* < 0.005; SpO_2_: Rho = −0.36, *p* < 0.005) and AT (AHI: Rho = 0.37, *p* < 0.005; SpO_2_: Rho = −0.35, *p* < 0.005) were associated with AHI and SpO_2_ while there was no correlation with SCF (AHI: Rho = 0.08, *p* = 0.43; SpO_2_: Rho = −0.01, *p* = 0.99). In separate multivariate models each including age and BMI, HLLV (*p* < 0.005) and NC (*p* < 0.05), but not IAF (*p* = 0.47), AT (*p* = 0.48) nor SCF (*p* = 0.84), were associated with AHI.

In a full model including age, BMI, HLLV and NC, both HLLV (partial R^2^ = 9%, *p* = 0.001) and NC (partial R^2^ = 7%, *p* = 0.004) were independent predictors of AHI, such that a 100-cm^3^ increase in HLLV and 1-cm increase in NC were independently associated with mean increases in AHI of 15 and 9%, respectively (Table 2).

**Table 2:** Most significant anthropometric predictors of AHI, SpO_2_, and ODI according to multiple linear regression analyses.

Predictors	AHI (log. values)	SpO_2_ (%)	ODI (log. values)
Age (years)	0.010 (0.004)	−0.123 (0.037)	0.007 (0.004)
	*p* = 0.012*	*p* = 0.002*	*p* = 0.073

BMI (kg/m^2^)	0.012 (0.006)	−0.161 (0.056)	0.015 (0.006)
	*p* = 0.043*	*p* = 0.005*	*p* = 0.013*

NC (cm)	0.039 (0.013)	−0.321 (0.126)	0.033 (0.014)
	*p* = 0.004*	*p* = 0.012*	*p* = 0.019*

HLLV (100 cm^3^)	0.060 (0.018)	−0.347 (0.17)	0.052 (0.018)
	*p* = 0.001*	*p* = 0.044*	*p* = 0.005*

Intercept	−1.775 (0.561)	120.312 (5.405)	−1.473 (0.578)

Explained variance	*R*^2^ = 0.310*	*R*^2^ = 0.297*	*R*^2^ = 0.282*

Beta coefficients in each cell are reported as mean values with SE and significance (*p < 0.05).

*Beta coefficient of VLES is expressed by increase of 100 cm^3^ of VLES*.

*Prior to regression analysis, AHI and ODI values were expressed in a logarithmic scale, e.g., LOG_10_(1 + AHI)/LOG_10_(1 + ODI), to approximate a Gaussian distribution*.

*AHI, apnea/hypopnea index; BMI, body mass index; HLLV, hepatic left liver volume; NC, neck circumference; SpO_2_, mean nocturnal O_2_ saturation; ODI, oxygen desaturation index*.

In a full model including age, BMI, HLLV, and NC, HLLV (partial R^2^ = 9%, *p* = 0.005) was an independent predictor of ODI, such that a 100-cm^3^ increase in HLLV was independently associated with an average 13% increase in ODI (Table 2).

**Figure 3:**
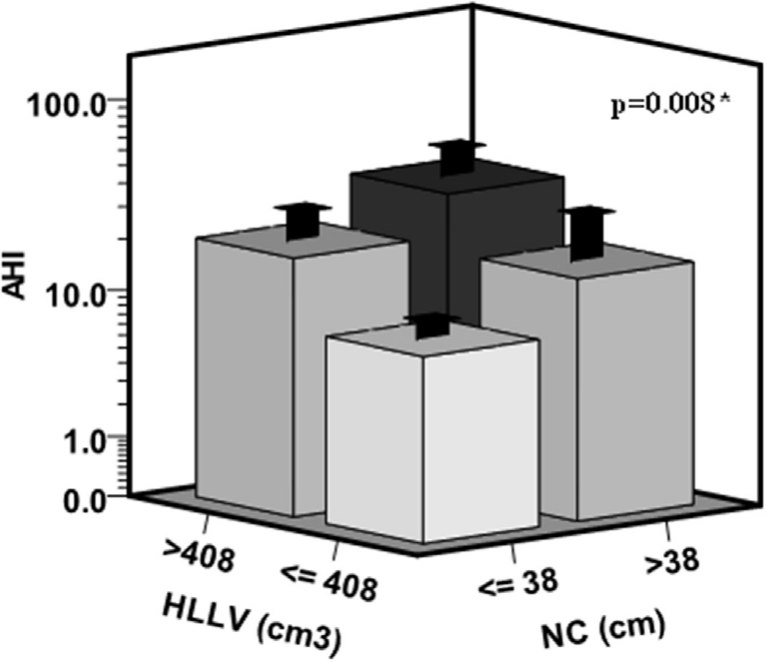
Cumulative effects of hepatic left lobe volume (HLLV) and neck circumference (NC) on the apnea/hypopnea index (AHI). Subjects were categorized in four subgroups according to the median values of HLLV (=408 cm^3^) and NC (=38 cm) in the whole cohort. The variances of AHI values were not significantly different among the four subgroups by the Levene’s test (*p* = 0.07). *p < 0.05 by ANOVA.

To further illustrate the independent effects of HLLV and NC on AHI, we categorized subjects in four subgroups according to the median values of HLLV and NC (Figure 3) and women with a HLLV > 408 cm^3^ and NC > 38 (*n* = 14, median AHI = 15) had higher AHI compared to those with an HLLV < 408 cm^3^ and NC < 38 cm (*n* = 25, median AHI = 5, *p* < 0.005). Similarly, NC (partial *R*^2^ = 5%, *p* < 0.05) and HLLV (partial *R*^2^ = 3%, *p* < 0.05) were independent predictors of SpO_2_ after adjustment for age and BMI (Table 2).

### Predictors of OSAHS Diagnosis

**Figure 4:**
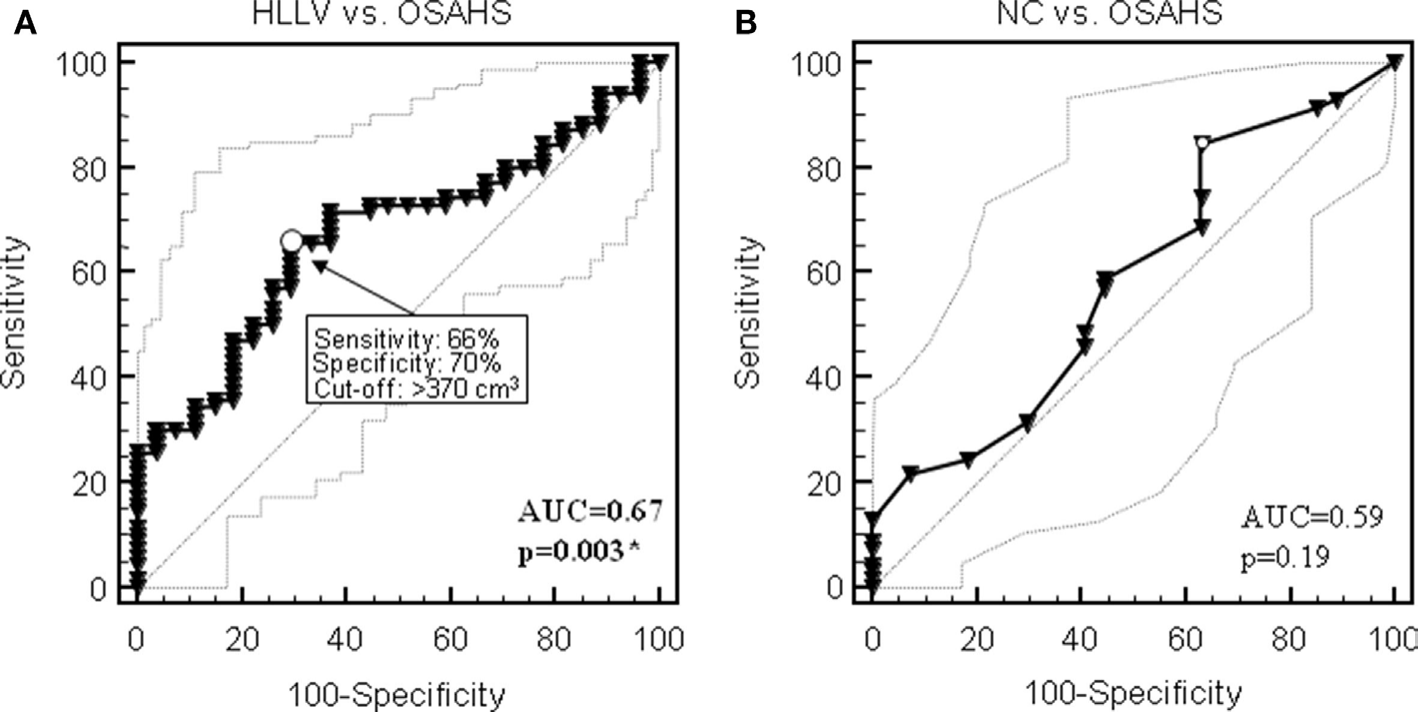
Receiver operating characteristic (ROC) curves for the diagnostic performance of hepatic left lobe volume (HLLV) **(A)** and neck circumference (NC) **(B)** to identify obstructive sleep apnea syndrome (OSAHS) in the cohort of 97 obese patients. Thin lines show 95% confidence intervals; arrows point at the optimal cutoff as defined by the Younden index for diagnostic sensitivity and specificity. AUC, area under the curve. *p < 0.05 by ROC curve analysis.

In the whole cohort of 97 women, HLLV was a parameter significantly associated with OSAHS by ROC curve analysis (AUC = 0.67, 95% CI: 0.57–0.76, *p* < 0.005) (Figure 4A). A cutoff value of 370 cm^3^ had a sensitivity of 66% (95% CI: 53–77%), a specificity of 70% (95% CI: 50–86%), and positive and negative predictive values (NPVs) of 85 and 44%, respectively, in identifying subjects with OSAHS. Conversely, NC did not discriminate between subjects with or without OSAHS (AUC = 0.59, *p* = 0.19) (Figure 4B). Considering the full logistic model, HLLV (*p* < 0.05, OR = 1.003, 95% CI: 1.001–1.006), but not NC, (*p* = 0.09) was the only independent predictor of OSAHS diagnosis after adjustment for age (*p* < 0.005) and BMI (*p* = 0.86) in the multivariate analysis. According to the results of the logistic analysis, a predicted probability of OSAHS (AHI ≥ 5) was calculated on the basis of age, BMI, NC, and HLLV applying the following formula: 1/[1 + exp(−SCORE)], where SCORE = −9.806 + age (years) × 0.075 + BMI (kg/m^2^) × 0.007 + NC (cm) × 0.155 + HLLV (cm^3^) × 0.003. The predicted probability of OSAHS achieved the highest performance in the classification of OSAHS (AUC = 0.79, 95% CI: 0.70–0.87, *p* < 0.005) and a cut-off value equal to 0.70 yielded to the best diagnostic results in terms of combined sensitivity (76%), specificity (89%), NPV (59%), positive predictive value (95%), and accuracy (79%). Among subjects with a predicted probability of OSAHS ≥ 0.70, only 3 (5%) did not actually present OSAHS (false positive patients) while among the subjects with a predicted probability of OSAHS < 70%, 17 (41%) presented OSAHS (false negative patients). Results for each class of OSAHS severity are reported in Table 3. Of note, all patients with severe OSAHS were identified by this prediction model.

**Table 3:** Comparison between the predicted probability of obstructive sleep apnea–hypopnea syndrome (OSAHS) (calculated by multivariate logistic model which includes age, BMI, HLLV, NC) and the actual prevalence of OSAHS.

Predicted probability of OSAHS	No OSAHS [apnea/hypopnea index (AHI) < 5]	Mild OSAHS (AHI ≥ 5 and <15)	Moderate OSAHS (AHI: 15–30)	Severe OSAHS (AHI > 30)
<0.70	*N* = 24 (89%)	*N* = 13 (35%)	*N* = 4 (22%)	*N* = 0 (0%)
≥0.70	*N* = 3 (11%)	*N* = 24 (65%)	*N* = 14 (78%)	*N* = 15 (100%)

## Discussion

Obstructive sleep apnea–hypopnea syndrome and NAFLD are very common in patients with central obesity and MS (18). Regional fat distribution is different in each sex, with fat tending to accumulate primarily around the waist in men and around the hips in women; the predominance of central obesity in men partly explains the difference in the prevalence of OSAHS between the sexes. In the Wisconsin Sleep Study Cohort, women had a higher BMI than men at each level of respiratory disturbance index and less severe apnea at an equivalent degree of obesity (8, 32). Male patients with OSAHS have a greater amount of visceral adipose tissue by computed tomography as compared to BMI-matched men without sleep-disordered breathing, and visceral but not subcutaneous fat appears significantly correlated with indices of sleep apnea (33).

Age is another risk factor for OSAHS especially in women (8), probably due to the protective effect of female sex hormones before menopause. Bixler et al. in a epidemiologic study of OSAHS in women found that the prevalence was higher in postmenopausal women that in premenopausal women (3.9 vs 0.6%) (34).

Female subjects are referred less frequently to sleep clinics probably also because of gender-related symptom differences; women with OSAHS may refer atypical symptoms such as fatigue, headaches, mood disorders (32, 35).

In studies conducted in numerically limited populations with predominance of male subjects affected by overweight or mild and moderate obesity, higher values of BMI, waist circumference (or waist–hip ratio), neck circumference were almost always associated with the presence and severity of OSAHS (9, 36–39). However, anthropometric parameters appear inadequate in predicting the risk of OSAHS and the systematic use of polysomnography is recommended (40, 41). Waist circumference may not be reliable in severe obesity due to its imprecision and inability to palpate the iliac crest besides the confounding effect of cutaneous plications; in addition, it cannot distinguish between subcutaneous and intra-abdominal fat (IAF). In severe obesity, it was also found a weak association between AHI and excessive daytime sleepiness or other symptoms and signs of OSAHS especially in females. Obesity itself is a cause of poor subjective assessment of sleep quality and sleepiness (15, 42, 43).

Recognizing the risk factors and predictors of OSAHS may be of particular relevance in women. Indeed, women are more frequently addressed to bariatric surgery than men and patients with OSAHS are particularly vulnerable during anesthesia and sedation, and display an increased risk of developing respiratory and cardiopulmonary postoperative complications (44). Identifying anthropometric, clinical, and laboratory predictors of risk of OSAHS would be useful in clinical practice in order to avoid expensive investigations such as polysomnography and to select patients that should be submitted to specific diagnostic test.

Few studies have analyzed the prevalence of OSAHS in morbidly obese women; the most important risk factors identified were BMI, age, and menopausal status (42, 43, 45).

In our sample, OSAHS was found to be present in 72% of obese women with a BMI greater than 35. Approximately half of the patients had mild OSAHS, one quarter had moderate OSAHS and one quarter of patients manifested a severe form of obstructive sleep apnea. Patients with OSAHS were older and had a greater visceral fat estimated by ultrasound compared to patients without OSAHS, despite similar body weight, BMI, NC, and SCF. γ-GT, but not transaminase, levels positively correlated with OSAHS quantified by AHI index, suggesting that OSAHS could be an independent risk factor for NAFLD. Lack of association between AHI index and serum transaminases may depend on the limited statistical power of our study sample. However, liver enzymes alone are not sensitive enough to characterize NAFLD. In this regard, it should be noted that non-invasive liver biomarkers-based scores have been developed to evaluate the extent of liver injury, which are independently associated with the severity of nocturnal hypoxia (46, 47).

As expected, neck circumference (NC) and IAF (measured by ultrasound) correlated with AHI and ODI and with the severity of OSAHS. We also observed a positive relationship between HLLV and AHI. ODI correlated with HLLV in a positive fashion as well. Interestingly, HLLV showed a stricter correlation with parameters describing OSAHS than NC.

According to the ROC curve analysis, only HLLV but not NC did discriminate between subjects with or without OSAHS. Similarly, according to the logistic regression, when dichotomizing a continuous variable (AHI) in two groups, the statistic contribution of NC was lost.

These results confirm the role of liver enlargement as a marker of visceral adiposity, which may facilitate the identification of individuals who are at increased risk of OSAHS in addition to the other known risk factors of OSAHS such as age, BMI, and NC. Indeed, prediction logistic model, including age, BMI, NC, and HLLV, displayed a discriminative ability (evidenced by AUC value of 0.79). However, the NPV was too low (59%) to propose this model in a clinical setting. While it is useful to reduce the number of false positives to avoid unnecessary instrumental sleep studies, a relevant number of false negative results may lead to a failure to treat OSAHS with potential adverse consequences. Nevertheless, among the false negative, there were no patients with severe OSAHS (AHI > 30) indicating that the model can identify all patients affected by OSAHS requiring urgent and active treatment.

At present, we cannot establish whether OSHAS and NAFLD can influence each other in terms of occurrence and progression or if they are merely unrelated expressions of ectopic fat deposition. Future studies including HLLV and other non-invasive scores (46, 47) may be helpful in this regard.

In conclusion, the results of this study indicate that in severe obese women HLLV is a powerful and better anthropometric predictor of OSAHS than neck circumference, and we have identified a prediction logistic model based only on four parameters (age, BMI, NC, and HHLV), which is capable of predicting all severe forms of OSAHS requiring active treatment.

## Ethics Statement

Our study is a pure retrospective analysis of data collected from a cohort of patients who gave written consent to the treatment of their clinical data for purpose of research. No additional testing was performed beside those required for the evaluation of patients’ candidate to bariatric surgery. There are no ethical issues that may require approval by our EC.

## Author Contributions

GS and GQ conceived the study design, carried out data collection, and wrote the manuscript. LM performed cardiorespiratory overnight sleep study. PP performed the statistical analysis and drew the figures. SM performed ultrasound examination for measurement of hepatic left volume, subcutaneous and intra-abdominal fat. GC, PF, GS, JV, SB, and GC were involved in data interpretation and writing of the paper. AP and FS conceived the study design, contributed to data interpretation, and writing of the manuscript. All authors approved the final version.

## Conflict of Interest Statement

The authors declare that the research was conducted in the absence of any commercial or financial relationships that could be construed as a potential conflict of interest.

## Abbreviations

AHI, apnea/hypopnea index; ALP, alkaline phosphatase; ALT, alanine aminotransferase; AST, aspartate aminotransferase; AT, abdominal thickness; ATP-III, adult treatment panel; AUC, area under the curve; BMI, body mass index; CT, computed tomography; γ-GT, γ-glutamyltransferase; HDL, high-density lipoprotein; HLLV, hepatic left lobe volume; HOMA, homeostatic model assessment—insulin resistance; IAF, intra-abdominal fat; IDF, International Diabetes Federation; IQR, interquartile range; LDL, low-density lipoprotein; MS, metabolic syndrome; NAFLD, non-alcoholic fatty liver disease; NC, neck circumference; NPV, negative predictive value; ODI, oxygen desaturation index; OGTT, oral glucose tolerance test; OSAHS, obstructive sleep apnea–hypopnea syndrome; PPV, positive predictive value; R, Pearson correlation coefficient; Rho, Spearman correlation coefficient; ROC, receiver operating characteristic curve; SCF, subcutaneous fat; SpO_2_, nocturnal O_2_ saturation; TG, triglycerides.
